# A new multiple-locus variable-number tandem repeat analysis reveals different clusters for *Anaplasma phagocytophilum* circulating in domestic and wild ruminants

**DOI:** 10.1186/1756-3305-7-439

**Published:** 2014-09-16

**Authors:** Thibaud Dugat, Amélie Chastagner, Anne-Claire Lagrée, Elisabeth Petit, Benoît Durand, Simon Thierry, Fabien Corbière, Hélène Verheyden, Luc Chabanne, Xavier Bailly, Agnès Leblond, Gwenaël Vourc’h, Henri-Jean Boulouis, Renaud Maillard, Nadia Haddad

**Affiliations:** Université Paris-Est, Ecole Nationale Vétérinaire d’Alfort, UMR BIPAR ENVA Anses UPEC USC INRA, Maisons-Alfort, France; Institut national de la recherche agronomique, UR 346 Épidémiologie Animale, Saint Genès Champanelle, France; Université Paris-Est, Agence nationale de sécurité sanitaire de l’alimentation, de l’environnement et du travail, Laboratoire de Santé Animale, Unité d’Epidémiologie, Maisons-Alfort, France; Université Paris-Est, Agence nationale de sécurité sanitaire de l’alimentation de l’environnement et du travail, Laboratoire de Santé Animale, Unité des Zoonoses Bactériennes, Maisons-Alfort, France; Ecole Nationale Veterinaire de Toulouse, UMR 1225 Interactions Hotes Agents Pathogènes INRA-ENVT, Toulouse, France; Institut National de la Recherche Agronomique, UR 035 Comportement et Ecologie de la Faune Sauvage, Castanet-Tolosan, France; Université de Lyon VetAgroSup, Département des Animaux de compagnie, JE Hémopathogènes vectorisés, Marcy l’Etoile, France; Département Hippique, Université de Lyon VetAgroSup, Marcy l’Etoile, France; Ecole Nationale Veterinaire de Toulouse, Unité pathologie des ruminants, Toulouse, France

**Keywords:** *Anaplasma phagocytophilum*, MLVA, VNTR, Epidemiology, Reservoir, Wild ruminants, Domestic ruminants

## Abstract

**Background:**

*Anaplasma phagocytophilum* is a tick-borne intragranulocytic alpha-proteobacterium. It is the causative agent of tick-borne fever in ruminants, and of human granulocytic anaplasmosis in humans, two diseases which are becoming increasingly recognized in Europe and the USA. However, while several molecular typing tools have been developed over the last years, few of them are appropriate for in-depth exploration of the epidemiological cycle of this bacterium. Therefore we have developed a Multiple-Locus Variable number tandem repeat (VNTR) Analysis typing technique for *A. phagocytophilum*.

**Methods:**

Five VNTRs were selected based on the HZ human-derived strain genome, and were tested on the Webster human-derived strain and on 123 DNA samples: 67 from cattle, 7 from sheep, 15 from roe deer, 4 from red deer, 1 from a reindeer, 2 from horses, 1 from a dog, and 26 from ticks.

**Results:**

From these samples, we obtained 84 different profiles, with a diversity index of 0.96 (0.99 for vertebrate samples, *i.e.* without tick samples). Our technique confirmed that *A. phagocytophilum* from roe deer or domestic ruminants belong to two different clusters, while *A. phagocytophilum* from red deer and domestic ruminants locate within the same cluster, questioning the respective roles of roe *vs* red deer as reservoir hosts for domestic ruminant strains in Europe. As expected, greater diversity was obtained between rather than within cattle herds.

**Conclusions:**

Our technique has great potential to provide detailed information on *A. phagocytophilum* isolates, improving both epidemiological and phylogenic investigations, thereby helping in the development of relevant prevention and control measures.

**Electronic supplementary material:**

The online version of this article (doi:10.1186/1756-3305-7-439) contains supplementary material, which is available to authorized users.

## Background

It is now recognized that a pathogen’s ability to infect multiple, rather than just a single host species, is more the rule than the exception [1, 2]. However for each pathogenic species, it remains difficult to determine whether different animal species are randomly infected, or whether certain strains circulate in more or less distinct epidemiological cycles [3, 4]. Such knowledge is crucial in order to understand pathogenic epidemiological cycles and to formulate and implement relevant prevention and control measures. Nowadays, improved pathogen detection using molecular tools has opened up new opportunities to address these issues [5]. Molecular tools are invaluable for studying the circulation of isolates between hosts, as well as the respective roles of the different animal species involved.

Many pathogens transmitted by *Ixodes ricinus* typically exploit multiple different host species, as vector ticks can feed on diverse numbers of vertebrates [6]. This multiplicity of hosts is one of the main reasons why it is so difficult to identify the precise role of hosts involved in the epidemiological cycle(s) of certain tick-borne pathogens.

*Anaplasma phagocytophilum* is a tick-borne intragranulocytic alpha-proteobacterium [7], mainly transmitted by *I. ricinus* in Europe, by *I. scapularis* and *I. pacificus* in North America, and by *I. persulcatus* in Asia [8]. It infects a large range of hosts, including wild and domestic ruminants, dogs, horses, and rodents [2]. Animal infection has been detected in Europe, North and South America, Asia, and Africa [8–10].

*A. phagocytophilum* is the causative agent of granulocytic anaplasmosis in humans, horses, dogs and occasionally cats, and tick-borne fever (TBF) in domestic ruminants [8]. TBF is characterized by anorexia, agalactia, and in some cases, fetal abortion. The epidemiology of *A. phagocytophilum* infection differs greatly between the US and Europe. In the US, human granulocytic anaplasmosis (HGA) is an increasing public health problem (the CDC reported 1800 human cases in 2010, with a 0.7% fatality rate [11]), whereas no US cases of TBF have been described to date. Conversely, HGA appears to be more rare in Europe (even though the number of reported cases has increased during recent years, probably linked in part to improved surveillance [12, 13]), whereas many TBF cases have been described in both cattle and sheep, causing significant economic losses [14–16]. These discrepancies between continents within one species, suggest *A. phagocytophilum* variability between and within host species and between continents. Experimental studies support the hypothesis that different epidemiological contexts are associated with considerable strain variation [17, 18]: an American strain infectious for horses was not infectious for ruminants [19], whereas a European variant pathogenic for cattle did not cause any clinical disease in horses [20]. In the US, the Ap-ha variant, which is pathogenic for humans, can also infect both ruminants and mice under experimental conditions, whereas the Ap-Variant 1, which is not infectious for humans, can infect goats and deer, but not mice [21–24].

As *A. phagocytophilum* is not transmitted transovarially in ticks, it is thought to be maintained in vertebrate reservoir hosts. However, the reservoir hosts for European cattle and human strains have not yet been identified. Roe deer have been suspected as reservoir hosts for sheep strains in Norway [25]. However to date, their role is still unclear. Isolates from either roe deer, or sheep and cattle, belonged to different clusters based on *ankA* gene phylogeny [26], whereas examining the *groEL* locus [27] showed that isolates from domestic goats (*Capra hircus*), belonged to the same cluster as those from roe deer.

To understand the circulation of *A. phagocytophilum*, and to identify its reservoir hosts, the genetic diversity of this pathogen needs to be explored, requiring discriminant genetic markers. Currently, there are only a few molecular typing techniques available for *A. phagocytophilum,* which are currently unable to discriminate between all strains. These techniques include pulsed-field gel electrophoresis (PFGE) [28], and single locus typing [26, 27, 29]. Two potentially more discriminant multi-locus approaches have been developed. The first approach is based on sequencing four loci including three genes and a 16S-RNA locus segment, and the second, called MLST (Multi-Locus Sequence Typing), relies on the sequencing of seven loci [30–33] *.* MLST is well adapted for phylogenetic analysis, however, this technique does not have enough discriminatory power for traceability studies, which are better performed by Multiple Locus VNTR (Variable-Number Tandem Repeat) Analysis (MLVA) [34–37]. MLVA determines the number of tandemly repeat sequences at different polymorphic VNTR loci within a bacterial genome. In a growing number of prokaryotes, which includes those displaying low genetic heterogeneity, VNTR typing has proved to be discriminatory, simple and transferable, with excellent marker stability [38]. This type of epidemiological tool has now been used for many pathogenic bacteria, in particular for those bacteria with clinical impact on animals and/or with zoonotic impact [34, 39–43]. In a previous study, Bown *et al*. developed an MLVA technique for *A. phagocytophilum* [44] *,* based on intergenic microsatellite VNTRs. Paradoxically, this technique was too discriminatory for use in epidemiology, when analyzing epidemiological links between isolates from different host species. Moreover, as the basic VNTR units were too short to discriminate between the different alleles by gel electrophoresis, any results had to be systematically determined or confirmed by sequencing or capillary electrophoresis.

In the present study we have developed an MLVA technique that would be more appropriate for *A. phagocytophilum* typing. Our objective was to explore the genetic diversity of *A. phagocytophilum* obtained from domestic and wild ruminants, in order to better understand the circulation of this pathogen between these two populations. This information will aid in subsequent efforts to control cattle infection. To address our objective, five new intragenic VNTR minisatellites were selected. Using these new MLVA markers, 97 samples from different domestic and wild animal species, 26 tick samples, the HZ genome, and the Webster human strain were analyzed. Resultant sample diversity was then analyzed and compared to current *A. phagocytophilum* diversity data.

## Methods

### Ethics statement

The domestic animals used in this study met the definition of "farm animals" or "pets", which are not currently covered by French regulations (*Décret* 2013–118 dated the 1^st^ February 2013, from the French Ministry of Agriculture). The owners of the animals provided permission for studies using samples obtained from their animals.

**Figure 1:**
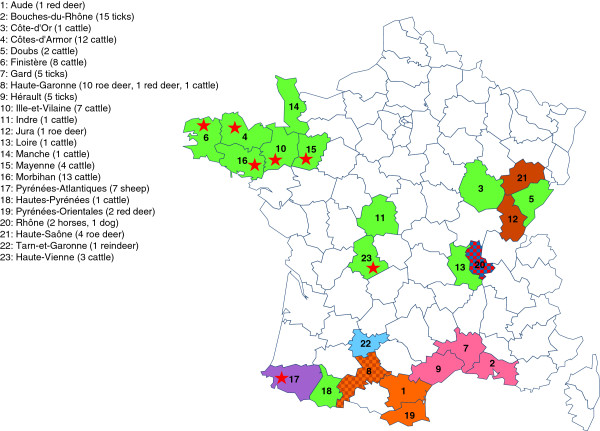
**Location of the French samples collected in this study.** Each color represents an animal host species: dark blue: horses; light blue: red deer; brown: dogs; green: cattle; orange: roe deer; pink: ticks; red: reindeer; yellow: sheep. French department names and numbers of samples per species in each French department are indicated in the legend. Red star: farm from which two or more samples were collected.

The wild ruminant (roe deer specimens from French departments 12 and 21, Figure 1) and red deer specimens (French departments 1, 8 and 19, Figure 1) were killed by gunshot by licensed hunters, during the legal hunting season, which fell under official annual hunting quotas delineated by the county prefect. Specific accreditation to collect such samples was granted by the National Agency for Hunting and Wildlife (ONCFS, accreditation number 2009–2014). Specimens were obtained from animals which had been killed for hunting reasons, and were not specifically killed for this study. Thus, ethics committee approval was not required for this part of the study.

The wild roe deer from French department 8 were live-captured during the winter of 2013 using large-scale drives and long-nets. Blood samples were obtained from the jugular vein, and all roe deer were released after sample collection. The UR 035 team was granted administrative authorization by the *Direction Départementale des Territoires* from department 8 to catch and inventory wild animals. Blood sampling was performed by team veterinarians who were authorized to perform animal experimentation.

### Samples and DNA extraction

DNA samples were obtained from animal blood or spleens, and from ticks. *A. phagocytophilum* was detected in these samples by PCR as previously described [45]. Hereafter, the term "samples" applies to field samples that gave positive PCR results for *A. phagocytophilum*. The HZ strain genome and 124 *A. phagocytophilum*-positive samples were analyzed: 67 cattle (*Bos taurus*), 7 sheep *(Ovis aries*), 15 roe deer (*Capreolus capreolus*), 4 red deer (*Cervus elaphus*), 1 reindeer (*Rangifer tarandus*), 2 horses (*Equus caballus*), 1 dog (*Canis lupus familiaris*), 26 ticks (19 *Rhipicephalus bursa*, 5 *Rhipicephalus sanguineus*, 1 *Rhipicephalus turanicus*, and 1 *Ixodes scapularis*), and the North American human Webster strain [46]. Sample characteristics are summarized in Table 1 (for more details see Additional file 1). DNA was subsequently extracted from all samples.

**Table 1:** **Host and geographical origin of the** ***A. phagocytophilum*** **-positive samples used for MLVA technique development**

Host	Geographical origin	Number of samples
Human strain HZ	USA, Minnesota	1
Human strain webster	USA, Wisconsin	1
Cattle (*Bos taurus*)	France (see Figure 1)	67
Sheep (*Ovis aries*)	France (7)	7
Roe deer (*Capreolus capreolus*)	France (8, 12 ,21)	15
Red deer (*Cervus elaphus*)	France (1, 8, 19)	4
Reindeer (*Rangifer tarandus*)	France (22)	1
Horse (*Equus caballus*)	France (20)	2
Dog (*Canis lupus familiaris*)	France (20)	1
Tick *(Rhipicephalus bursa*)	France (2, 7, 9)	19
Tick *(Rhipicephalus sanguineus*)	France (2, 7, 9)	5
Tick *(Rhipicephalus turanicus*)	France (9)	1
Tick *(Ixodes scapularis*)	USA	1

Between brackets: French department number (for the location of the French departments on the map of France, see Figure 1).

For DNA extraction from blood or ticks, the NucleoSpin® Blood QuickPure kit (Macherey-Nagel, Bethlehem, USA), or the NucleoSpin® Tissue kit (Macherey-Nagel) were used respectively, according to manufacturer’s instructions. DNA extracts were then stored at -20°C prior to testing.

### Computer analysis of repetitive DNA sequences for use as VNTR candidates

The genomic DNA sequence of the human *A. phagocytophilum* HZ strain (Reference Sequence NC_007797.1) [47] was screened for repetitive DNA sequences using the tandem repeats database developed by Le Flèche *et al*. [48].

The following criteria were applied to potential VNTR candidates: i/ Total length between 9 and 5000 bp; ii/ unit length between 9 and 500 bp; iii/ basic unit (BU) copy number between 1 and 2000; iv/ 70 to 100% BU identity.

BLASTN analysis of the repeat sequences excluded VNTRs present in other available genomes, especially those present in other *Anaplasmataceae*.

### Primer design for VNTR candidates

The tandem repeats database described by Le Flèche *et al.* [48] also provided 500 bp of flanking sequences both upstream and downstream of each VNTR candidate locus. BLASTN analysis of these flanking sequences was used to design forward and reverse primers. Primers were designed ensuring that no annealing would occur with; i) other *A. phagocytophilum* genome regions, ii) other available pathogen genomes and iii) available host genomes (*i.e*. human, both wild and domestic ruminants, canine, equine, rodent and tick), iv) the paired primer, or itself. Each VNTR candidate was designated as an APV (*Anaplasmaphagocytophilum* VNTR), followed by a letter. The location of each APV in the *A. phagocytophilum* genome is described as the ‘locus’. An ‘allele’ corresponds to a given number of repeat units for a particular APV or locus. APV characteristics are summarized in Table 2.

**Table 2:** **Characteristics of the selected APVs and the corresponding forward and reverse primers**

VNTR name	BU Length (bp)	Genome localization (HZ strain)	Gene	Primer Sequence 5′ - > 3′	Allele size range (bp)	Allele size range (BU)	Number of allele
APV-A	201	28845-29712	*APH_0032*	F TGTAAGCAAGCACCCAACGCGAA	130-865	0,5-4,5	6
				R GCCAGAATCGCAACACACTGACG			
APV-B	114	53792-54393	*rpe*	F GGGGGTATGACGAGTGTGGTAGCAA	0*-945	0*-9	10
				R CCTTACTGCACACCGTACACGCAAA			
APV-C	189	340359-340834	*APH_0351*	F CCTACGGGGTGTCTTGCGTCCTA	90-1400	0,5-7,5	10
				R CTGCGCGAGTTTATGTGCAACT			
APV-D	123	376959-377498	*virB6-3*	F ATAGTGTGCAAGGCGCTAGTAATG	355-750	3-6	5
				R TGTCGGACTATGCTTTTCACCATT			
APV-E	15	214596-214852	*APH_0215*	F CGACCTATGATCGCAGTGTA	5-925	0,5-62	14
				R GTAGCAAGGTAACCACTACCA			

F: forward; R: reverse.

* absence of VNTR.

### Amplification, analysis and selection of VNTR candidates

Protocol optimization was carried out on five different samples, and the ability of each VNTR to explore diversity was then tested on all samples. VNTR amplification was conducted in a volume of 25 μl, containing 5 μl purified DNA, 4 μl 5X high fidelity amplification buffer, 200 μM each dNTP, 0.5 μM each primer (Eurofins MWG Operon, Ebersberg, Germany) and 0.4 units of Phusion DNA polymerase (Fisher Scientific, Waltham, USA). An initial denaturation step at 98°C for 30 s was followed by the subsequent thermocycling protocol: DNA was denatured for 10 s at 98°C, and primers were annealed for 30 s at the optimal temperature (56°C for APVA, APVB, APVC and APVD, and 58°C for APV-E), and extended at 72°C for 1 min. After 35 cycles, there was a final extension step at 72°C for 10 min.

PCR products were separated by electrophoresis on 1% SeaKem LE agarose gels (Ozyme, Saint Quentin en Yvelines, France) in TBE buffer (Lonza, Basel, Switzerland), and stained with ethidium bromide for imaging.

### Sequencing

PCR products (20 μl) were sequenced by Sanger sequencing (Eurofins MWG Operon) to characterize the sequence of each new allele. Results were analyzed using Bioedit software version 7.2.5 (Ibis Biosciences, Carlsbad, USA).

### GenBank accession numbers

For each VNTR, the alleles are listed in order.

[GenBank: KM216273 - KM216278]

APVA_4.5rep; APVA_3rep; APVA_2rep; APVA_1.5rep; APVA_1rep; APVA_0.5rep

[GenBank: KM216279 - KM216287]

APVB_9rep; APVB_8rep; APVB_7rep; APVB_6rep; APVB_5rep; APVB_4rep; APVB_3.5rep; APVB_2.5rep; APVB_2rep

[GenBank: M216288 - KM216296]

APVC_7.5rep; APVC_5rep; APVC_4rep; APVC_3.5; APVC_3rep; APVC_2rep; APVC_1.5rep; APVC_0.7rep; APVC_0.5rep

[GenBank: KM216297 - KM216300]

APVD_3rep; APVD_4rep; APVD_5rep; APVD_6rep

[GenBank: KM216301 - KM216311]

APVE_45rep; APVE_32rep; APVE_26rep; APVE_23rep; APVE_21rep; APVE_18rep; APVE_15rep; APVE_11rep; APVE_7rep; APVE_4rep; APVE_0.5rep

### Data analysis

#### Discriminatory power

The Hunter and Gaston discrimination index (DI) was used to evaluate the discriminatory power of the selected APVs, [49], as recommended by the European Society of Clinical Microbiology and Infectious Diseases Study Group on Epidemiological Markers [50]. This index measures the probability that two samples or strains, randomly chosen, will have different types. It is defined by:


N: number of samples or strains; S: total number of profiles or alleles and n_j_: number of samples or strains with the profile or allele j. Polymorphism rate is considered high when the index is higher than 95% [51].

#### Diversity of MLVA profiles obtained for cattle from the same herd *vs*cattle from different herds

The Fisher’s exact test was used to compare the proportion of *A. phagocytophilum* from cattle sharing the same profile among two groups: animals originating from distinct herds (one bovine sample per herd), and animals having common origins (at least two bovine per herd). Statistical analysis was conducted at 95% confidence level. A p value less than 0.05 was considered statistically significant.

#### Similarity of MLVA profiles obtained within the same species

The similarities of MLVA profiles obtained from the same animal species were analyzed using a bootstrap method as previously described [52]. Only animal species for which two or more MLVA profiles had been observed were taken into account. The null hypothesis was that two pairs of MLVA profiles obtained from the same species, should be as similar as two pairs of MLVA profiles obtained from different species. The distance between two MLVA profiles was the number of VNTRs with differential numbers of repeats (distances thus varied between 0 and 5). The analysis was focused on the average of within-species mean distances between MLVA profiles. The observed value of this statistic of interest was first computed. A resampling procedure was then used to simulate bootstrap samples from the data, under the null hypothesis. The resampling procedure was based on an adjacency matrix that links species (matrix rows) and MLVA profiles (matrix columns). Each bootstrap sample was obtained by generating random permutations of the matrix column headers (the MLVA profiles). This procedure guarantees that the marginal sums of the adjacency matrix are constant (i.e. number of profiles per species, number of species per profile), whereas the profiles are randomly redistributed. Ten thousand bootstrap samples were thus generated and, for each of these, the statistic of interest was calculated. The corresponding distribution was finally examined to determine the empirical p-value of the null hypothesis test: this p-value was the proportion of the samples (simulated under the null hypothesis) for which the statistic of interest was below the actual value (computed from the data).

#### Cluster analysis

MLVA clustering was performed using the BioNumerics software package version 6.6 (Applied-Maths, Sint-Martens-Latem, Belgium), with the categorical distance coefficient and UPGMA (Un-weighted Pair Group Method with Arithmetic mean) clustering method. Data were analyzed as a character dataset. Based on this categorical distance coefficient analysis, the Minimum Spanning Tree (MST) graphing algorithm was used to represent the relationships between strains. The priority rule for constructing MSTs was set so that the type which had the highest number of single-locus variants would be linked first. A cut-off value of maximum differences of 1 VNTR was applied to define clonal complexes under the MST method.

## Results

### VNTR selection

Using the tandem repeats database developed by Le Flèche *et al.* [48], we pre-selected 721 VNTR candidates according to the criteria described above. APV candidates corresponding to the selection criteria were further tested by evaluating their polymorphism. Following amplification optimization (amplification cycle and temperature gradient modifications), only those VNTRs that were amplified and appeared to be polymorphic when tested on five of our samples, were then selected and used in this study. These VNTRs corresponded to those listed as APV-A, APV-B, APV-C, APV-D and APV-E. Except for APV-E (15 bp), all VNTR BUs are longer than 100 bp. The VNTR names, BU lengths, primer sequences, gene and genome localization, and allele size ranges are shown in Table 2.

### Specificity of the MLVA technique

*In silico* analysis of all selected primers in BLASTN confirmed that they were unable to hybridize with either the genomes of other known animal pathogens, or those of microorganisms found in ticks. This feature was experimentally confirmed, as no PCR amplification was observed for DNA extracts from these microorganisms.

### Allele sequencing

The length of each allele was confirmed by sequencing. The different allele sizes are detailed in Table 2.

### Discriminatory power

The discriminatory power of each APV locus was estimated from the genetic DI values based on the numbers of alleles and their frequencies in different samples. The high individual DI values for the 124 samples, as well as the HZ genome included in our study, reflect their excellent potential as markers of genetic diversity: 0.80 for APV-A, 0.74 for APV-B, 0.74 for APV-C, 0.50 for APV-D, and 0.87 for APV-E (and without tick samples: 0.76 for APV-A, 0.69 for APV-B, 0.76 for APV-C, 0.60 for APV-D, and 0.88 for APV-E). Moreover, the global DI value, when combining the five APVs, was 0.96 (and 0.99 without tick samples). In total, we observed 84 different profiles for 125 samples.

#### Cattle samples

Sixty-seven cattle samples tested positive for *A. phagocytophilum*. Of these, twenty-one were obtained from seven farms located in various French departments, indicated in Figure 1: three samples from a Côtes-d’Armor farm (department 4 in Figure 1), two samples from a Finistère farm (department 6), three samples from a Haute-Vienne farm (department 23), four samples from an Ille-et-Vilaine farm (department 10), three samples from a Mayenne farm (department 15), and six samples from two Morbihan farms (three samples per farm, department 16). Different samples from the same farms harbored similar or identical profiles. For this set of 21 samples, 38% (n = 8) shared their profile with at least one other sample. For the 46 remaining cattle samples from different herds, our technique identified 44 distinct profiles. Within this group, 7% (n = 3) shared their profile with at least one other sample, the proportion of which was significantly higher for the first group (≥2 cattle per farm) than for the second group (1 cow per farm) (p = 0.003). This difference was also significant for cattle samples from Bretagne (French departments 4, 6, 10, 14, 15 and 16), where the majority of farms with multiple samples were located (exact Fisher’s test, p = 0.01347).

#### Other samples

**Figure 2:**
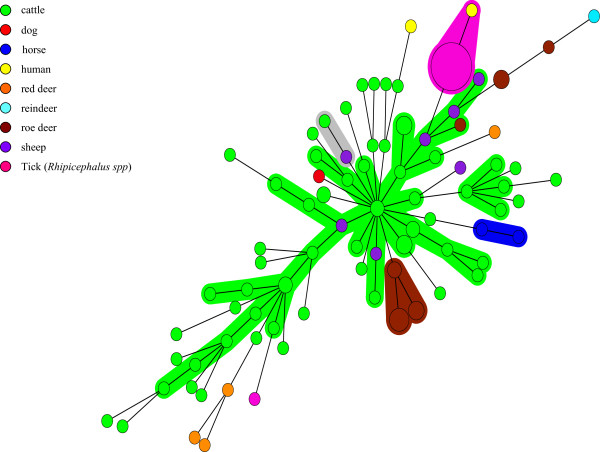
**Minimum spanning tree for the five VNTRs of 125** ***A. phagocytophilum*** **samples, strain and genome.** Each circle represents a unique genotype. The diameter of each circle corresponds to the number of field samples with the same genotype. Genotypes connected by a shaded background and tick lines differ by a maximum of one of the five VNTR markers, and could be considered as a "clonal complex". Regular connecting lines represent two loci differences; thin interrupted lines represent four or more differences. The length of each branch is proportional to the number of differences. Each host is represented by a specific color in the circle.

Two different but very similar profiles were observed for the two horse samples from the same area, as only the APV-E allele varied between those samples. Seven different profiles were obtained for seven sheep samples from one Pyrénées-Atlantiques herd. Three different but close profiles were observed for 10 roe deer samples from French department 8. These three MLVA profiles differed only by their APV-C alleles. Four different profiles were obtained for five roe deer samples from Franche-Comté (French departments 12 and 21), which differed according to their APV-A, APV-C and APV-E alleles. And finally, four different profiles were obtained from four red deer samples (French departments (1, 8 and 19). According to Figure 2, these profiles are very close to those from both cattle and sheep.

All 25 tick samples from the three different French departments of Camargue (15 from the Bouches-du-Rhône, five from Gard and five from the Hérault department; Figure 1, Table 1 and Additional file 2), a 250-km^2^ area in southern France, all harbored the same profile. Conversely, the tick sample aph1 (North American strain) provided a profile that was different from that observed in Camargue ticks.

### Cluster analysis

A total of 125 samples (84 VNTR profiles) were utilized for this analysis. Bootstrap analysis showed that MLVA profiles obtained from the same host species were significantly closer than the null hypothesis (according to which, the average distance between MLVA profiles is the same between species and within species) (p < 0.0001). MST analysis clearly differentiated *A. phagocytophilum* MLVA profiles from cattle, sheep, and red deer, compared to *A. phagocytophilum* from roe deer, positioning them in two different clusters (Figure 2). Moreover, *A. phagocytophilum* from domestic ruminants shared alleles at the APV-B locus with red deer, but not with *A. phagocytophilum* from roe deer.

## Discussion

MLVA has become a popular typing approach for studying the epidemiology of pathogenic agents, due to several advantages: high discriminatory power, robustness, repeatability, inter-laboratory portability and speed [53, 54]. In this study, we developed an MLVA technique for *A. phagocytophilum*, an intracellular tick-borne bacterium which can be isolated from both humans and various animal species. To the best of our knowledge, our work is the first study to be performed in France which typed *A. phagocytophium* from domestic and wild ruminants. Our MLVA technique has been shown to be effective for the typing of human, cattle, sheep, roe deer, red deer, reindeer, dog, horse, and tick isolates. This technique is based on five VNTRs with a global Hunter and Gaston discrimination index of 0.96 (84 profiles/125 samples), or 0.99 without tick samples, emphasizing its high discriminatory power.

In a previous study, Bown *et al.* developed an MLVA technique based on intergenic minisatellite VNTRs, but paradoxically, their method was too discriminatory [44]. The 20 strains tested in their study shared only a few alleles at each of the four loci (VNTR1 and 4: 17 different alleles/20 isolates; VNTR5 and 8: 14 different alleles/20 isolates, where each given allele was either shared by one isolate – a majority of cases – or up to 5 isolates). For this reason, their technique does not seem well adapted to studying either *A. phagocytophilum* transmission between species, or the circulation of isolates within cattle herds, or between wild animals – such as rodents – and domestic ruminants. This was an additional reason why we based our MLVA technique on intragenic minisatellite VNTRs, which are probably under higher selective pressure, and could be less variable, than those selected by Bown *et al.* as already shown for other bacteria [55]. Our results confirmed this hypothesis, as we obtained, among the 125 samples tested, six different alleles for APV-A, 10 different alleles for APV-B and APV-C, five different alleles for APV-D, and 14 different alleles for APV-E. Moreover, our technique appeared to efficiently discriminate related strains isolated from the same area and/or within the same animal species, and even regardless of the species. The two horse samples, both collected in Lyon (France), shared four alleles. APV-E, which is the most variable of our markers, was the only VNTR that differentiated between these two samples, differing by 30 nucleotides. In addition, whereas cattle samples isolated from different herds gave 44 profiles from 45 samples, samples obtained from cattle belonging to the same herds gave identical or very similar MLVA profiles. These results were confirmed even for cattle originating from the same region (Bretagne), which therefore excluded any links between geographical location and profile distribution. Conversely, we obtained highly diverse MLVA profiles within the sheep herd that was tested. If these divergent results were confirmed in more herds under similar conditions, they could perhaps be explained by differences in farming methods between the two animal species. For example, whereas cattle are confined to small pastures, sheep have extensive grazing areas, and are also subject to seasonal transhumance across a variety of territories, thus increasing their likelihood of exposure to different ticks, and consequently, diverse *A. phagocytophilum* strains. In addition, cattle are not persistently infected, in contrast to sheep [8]. Thus genetic shifts and/or inter-strain recombinations within the same sheep flock cannot be excluded, whereas the isolates obtained for cattle belonging to the same farm are most likely possibly derived from the same parental strain. This last result suggests that *in vivo* variations could occur under the selective pressure of the host immune system. However, no variation was detected after eight passages of the Webster strain in HL-60 cells, corresponding to approximately one year of culture (results not shown), suggesting that the observed lack of VNTR variations could be due to the absence of *in vitro* selective factors. Unfortunately, as artificially infecting animals with *A. phagocytophilum* requires specific animal facilities, we could not confirm whether *in vivo* variations are likely to occur in the field.

Taken together, our results strongly suggest that this particular MLVA technique has a good concordance with epidemiological contexts. The in-depth resolution obtained will be of great value for studying *A. phagocytophilum* circulation within cattle, or between wildlife and domestic ruminants. Epidemiological studies with suitable samples could validate this assertion, as well as the applicability of our MLVA technique for such studies.

We also noticed that the APV-B VNTR was systematically different in roe deer from other ruminant species samples. As our sampling is limited, further analysis of additional samples is needed in order to confirm or not if this VNTR could be considered as a marker of roe deer strains.

Our results suggest that red deer are reservoir hosts for domestic ruminant strains, in contrast to roe deer. Nevertheless, this observation has to be taken with caution as we could only test four red deer samples that were all collected from the same region, which does not permit the examination of geographical effects. However, red deer are becoming increasingly suspected as having an important role in the epidemiological cycle of *A. phagocytophilum*, as reservoir hosts. Different arguments support this hypothesis. Firstly, sheep experimentally infected with *A. phagocytophilum* red deer isolates developed TBF symptoms [25]. Secondly, high prevalence of *A. phagocytophilum* infection has been detected in red deer in many European countries. [27, 56–61]. In France, 80% of red deer (28/35) recently tested by Zehnter *et al.* from one French region (Correze), were reported to be seropositive for *A. phagocytophilum* [62]. According to official hunting bag data (ONCFS: the French national agency for wildlife), the average red deer population has increased three-fold in France over a twenty year period, and is continuing to increase [63, 64]. Unfortunately, no data are available which could either confirm or deny whether a parallel increase in French BGA incidence has occurred during the same period.

Thus, to date, the circulation of isolates between wild and domestic ruminants still remains unclear. Within this context, our MLVA technique could be a very useful tool with which to address this problem. For a reliable approach, more wild and domestic animal samples originating from the same regions will be required.

In addition, our results suggest the existence of an alternative epidemiological cycle in the French Camargue, involving *Rhipicephalus* ticks as potential vectors.

In Europe, *A. phagocytophilum* is mainly transmitted by *I. ricinus*, but *A. phagocytophilum* DNA has also been detected in other tick genera, such as *Dermacentor* and *Rhipicephalus* [65, 66]. Nevertheless, the ability of those ticks to transmit *A. phagocytophilum* has not yet been proven. In contrast to *Rhipicephalus* ticks, *I. ricinus* is considered to be rare, or even absent in the Camargue [67]. It means that in this region, *A. phagocytophilum* is, at least predominantly, transmitted by another vector, which could be *Rhipicephalus* ticks.

Chastagner *et al*. have found only one *A. phagocytophilum* MLST genotype in *Rhipicephalus* ticks in the Camargue [33]. Using our MLVA technique on the same samples, we have shown that all presented the same profile. Moreover, the fact that one North American tick sample provided a profile that was completely different from the profile obtained from Camargue tick samples, confirms that our MLVA technique is effective in revealing diversity at the level of tick isolates. As the ticks were collected at different sites, over an area of 250 km^2^, our results suggest the circulation of one ecotype of *A. phagocytophilum* in the French Camargue, which is adapted to *Rhipicephalus* ticks, representing a very particular ecosystem. In order to test this hypothesis, the competence of *Rhipicephalus* ticks for *A. phagocytophilum* transmission should be explored. Using our MLVA technique, we would be able to determine which variant(s) are detected, and if they could be multiplied and transmitted by *Rhipicephalus* ticks. Typing a large number of Camargue samples collected from different animal species would be an excellent complementary method to further characterize the epidemiological cycle of *A. phagocytophilum* in this very distinct French region, and in particular, the role of *Rhipicephalus* ticks.

## Conclusions

In conclusion, our results potentially reveal the existence of at least two different epidemiological transmission cycles of *A. phagocytophilum,* as has been previously described in the United Kingdom [29]. The first cycle may involve red deer as reservoir hosts, and possibly domestic ruminants, as either accidental or longer-term hosts, whereas the second might involve roe deer. In addition, our study reveals the existence of a potentially alternative epidemiological cycle in the Camargue region, which could involve *Rhipicephalus* ticks as vectors. These hypotheses should be further explored, and the MLVA technique described here would be suitable for such studies. Our MLVA technique could also be used in order to compare the molecular profiles of *A. phagocytophilum* from different areas and/or countries, as has already been performed for *B. henselae* in our laboratory [52, 68, 69]. In the case of *A. phagocytophilum,* this would be an important contribution to the in-depth characterization of European epidemiological cycles, and aid in the comparison with the rather different cycles identified in the USA.

## Electronic supplementary material

Additional file 1: **Origin (host species, herd, and geographical area), and year of sampling for the** ***A. phagocytophilum*** **field samples, strain and genome used in this study.** (XLS 34 KB)

Additional file 2: **MLVA profiles of** ***A. phagocytophilum*** **field samples, strain and genome used in this study.** (XLS 36 KB)

## Abbreviations

APV: *Anaplasma phagocytophilum* VNTR
BGA: Bovine granulocytic anaplasmosis
BLAST: Basic local alignment search tool
BU: Basic unit
CC: Clonal complex
CDC: Centers for disease control and prevention
DI: Diversity index
HGA: Human granulocytic anaplasmosis
MLST: Multi-locus sequence typing
MLVA: Multiple-locus variable-number tandem repeat analysis
MST: Minimum spanning tree
ONCFS: French national agency for wildlife
PFGE: Pulsed-field gel electrophoresis
SLV: Single-locus variant
TBF: Tick-borne fever
UPGMA: Unweighted pair group method with arithmetic mean
VNTR: Variable number tandem repeat.
